# Surgical management of giant cell arteritis of the proximal aorta

**DOI:** 10.1016/j.xjon.2024.08.017

**Published:** 2024-09-06

**Authors:** Motahar Hosseini, Alberto Pochettino, Joseph A. Dearani, Alejandra Castro-Varela, Hartzell V. Schaff, Katherine S. King, Richard C. Daly, Kevin L. Greason, Juan A. Crestanello, Gabor Bagameri, Nishant Saran

**Affiliations:** a Department of Cardiovascular Surgery, Mayo Clinic, Rochester, Minn; b Department of Surgery, Mayo Clinic, Rochester, Minn; c Department of Quantitative Health Sciences, Mayo Clinic, Rochester, Minn

**Keywords:** giant cell arteritis, aortic reintervention, aneurysm, survival

## Abstract

**Objective:**

Giant cell arteritis (GCA) may present as proximal aortic pathology requiring surgical intervention. We present our experience with surgical management of GCA in patients presenting with proximal aortic disease.

**Methods:**

From January 1993 to May 2020, 184 adult patients were diagnosed with GCA on histopathology after undergoing cardiac surgery. Survival was estimated with Kaplan-Meier method. Reoperation rates were estimated with cumulative incidence accounting for competing risks of death.

**Results:**

The most common indication for surgery was ascending aortic aneurysm (n = 179, 97.3%). Stroke occurred in 6 (3.3%), pneumonia in 8 (4.4%), and dialysis in 3 (1.6%) patients. Multivariable analysis found advanced age (hazard ratio [HR], 1.054; 95% confidence interval [CI], 1.026-1.082, *P* < .001), recent heart failure (HR, 1.890; 95% CI, 1.016-3.516, *P* = .04), peripheral vascular disease (HR, 2.229; 95% CI, 1.458-3.624, *P* < .001), and cerebrovascular disease (HR, 1.762; 95% CI, 1.035-3.000, *P* = .03) as predictors of late mortality. Median follow-up was 13.7 years, and 30-day mortality was 1.5%. Nineteen patients underwent 24 aortic reinterventions including aortic arch reconstruction (n = 4), descending thoracic aorta aneurysm repair (n = 8), thoracoabdominal aortic aneurysm repair (n = 11), and pseudoaneurysm repair (n = 1). Rate of reintervention on the aorta was 3.9% (95% CI, 1.9%-8.1%), 7.1% (95% CI, 4.1%-12.3%), 12.8% (95% CI, 8.3%-19.6%), and 12.8% (95% CI, 8.3%-19.6%) at 1, 5, 10, and 15 years, respectively.

**Conclusions:**

Surgery in patients with GCA can be performed with acceptable early and late outcomes. Advancing age, heart failure, peripheral vascular disease, and cerebrovascular disease are risk factors for worse survival. Postoperative surveillance is important as need for aortic reintervention is not uncommon.

**Figure:**
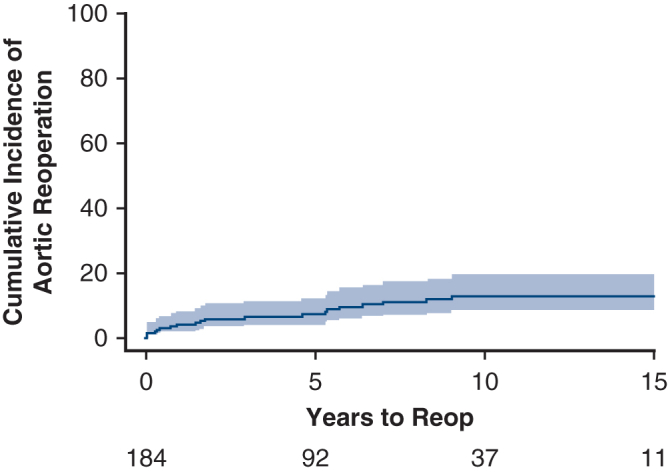
Cumulative incidence of aortic reoperation.

**Central Message**

Surgery for giant cell arteritis of the proximal aorta has satisfactory outcomes. Postoperative surveillance should be considered because aortic reintervention is not uncommon.

**Perspective**

Giant cell arteritis can manifest as proximal aortic pathology, necessitating surgical intervention. We provide long-term data on cardiac and aortic reoperations, underscoring the critical importance of follow-up care for these patients.

Giant cell arteritis (GCA) is a systemic disease common among elderly patients, affecting 17 cases per 100,000 persons per year.^1^ The most common site of involvement is the external carotid artery and its branches, followed by the large vessel variant, where upper extremities and aortic branch vessels are involved.^2^ In 10% to 40% of patients with GCA, the aorta is involved, with GCA being the most common systemic vasculitis to involve the aorta.^2^, ^3^, ^4^, ^5^, ^6^ GCA of the aorta can manifest with cranial symptoms such as headache, jaw claudication, or visual changes and can be associated with polymyalgia rheumatica (PMR).^2^^,^^7^ Alternatively, it can present as an asymptomatic isolated aortic aneurysm found incidentally on histopathology after aneurysm resection.^8^, ^9^, ^10^ This represents a variant of GCA, especially in patients older than 50 years of age.^9^^,^^10^

Data on the surgical management of these patients remain sparse and are limited by short series of patients.^4^^,^^8^ We, therefore, sought to review our institutional experience of patients who underwent ascending aorta surgery and were found to have histopathologically proven GCA from the aortic specimen. We further aimed to evaluate the surgical outcomes and reoperation rates in such patients.

## Methods

### Patients

We included consecutive adult patients (n = 184) who underwent ascending aorta surgery from April 1993 to May 2020 and had a confirmed diagnosis of GCA on the basis of histopathologic examination of the surgical aortic specimen. Patients with infectious aortitis, Takayasu arteritis, and other noninfectious aortitis were not included in the study. The study was deemed exempt by the Mayo Clinic Institutional Review Board (institutional review board no. 21-001978, March 5, 2021). Baseline patient characteristics were obtained from review of electronic medical records and were reported on the basis of definitions set forth by the Society of Thoracic Surgeons Adult Cardiac Database (STS Adult). A retrospective review of the surgical database and patient medical records, including operative notes, was performed.

### Statistical Analysis

Continuous data are described as median (quartile 1, quartile 3), and categorical data are described as count (percentage). Kaplan-Meier method was used to assess survival, and the reverse Kaplan-Meier method was implemented to assess follow-up availability. Proportional hazards regression was used to identify baseline and operative characteristics associated with survival. The survival of the cohort was compared with the White population in Minnesota on the basis of matched age and sex of the cohort. For the risk of reoperation, cumulative incidence estimates are provided, which account for the competing risk of death. The time to first reoperation was assessed and other reoperations were not treated as a competing risk. Aorta related reoperation was defined as any reoperation involving segments distal to the ascending aorta including anastomotic pseudoaneurysm. Cardiac reoperation was defined as any reoperation that did not involve the aorta.

## Results

### Baseline Characteristics

Demographics and significant comorbidities are presented in Table 1. Median age was 74 years (interquartile range [IQR], 70, 77) and 131 (71.2%) patients were female. Fifty-two (33.8%) patients were in New York Heart Association class III or IV. Hypertension and peripheral arterial disease were present in 141 (77.0%) and 38 (20.7%) patients, respectively. Twenty-four (13%) patients had known GCA before surgery. Twenty-three (15.2%) patients had a history of previous positive temporal artery biopsy for GCA. Additional diagnoses in these patients included 45 (30.2%) patients with a diagnosis of PMR and 7 (4.7%) patients with a diagnosis of rheumatoid arthritis. We found 41 (23%) patients who were on steroid therapy before index surgery either for the diagnosis of GCA or alternate reasons such as PMR and rheumatoid arthritis. On histopathology, there was a granulomatous inflammatory infiltrate mostly involving the media as well as lymphoplasmacytic infiltrate, consistent with previous published data.^2^ No aortic valve cusp tissue involvement with the inflammatory process was documented in patients who underwent aortic valve resection.

**Table 1:** Baseline preoperative characteristics in patients with giant cell arteritis

Characteristics	Number missing	Overall (N = 184)
Age, y	0	74 (70, 77)
Sex: female	0	131 (71.2%)
Body mass index, kg/m^2^	0	26 (23, 30)
Aortic aneurysm diagnosis	0	184 (100.0%)
Polymyalgia rheumatica	35	45 (30.2%)
Rheumatoid arthritis	34	7 (4.7%)
Steroid use	6	41 (23.0%)
NHYA classification III/IV	30	52 (33.8%)
Coronary artery disease	0	72 (39.1%)
Congestive heart failure within 2 wk	1	17 (9.3%)
Dyslipidemia	1	113 (61.7%)
Diabetes	1	14 (7.7%)
Hypertension	1	141 (77.0%)
Peripheral vascular disease	1	38 (20.8%)
Cerebrovascular disease	1	28 (15.3%)
Renal failure (dialysis or creatine >2 mg/dL)	1	1 (0.5%)
Dialysis	1	0 (0.0%)
Known giant cell before surgery	5	24 (13.4%)
Giant cell arteritis found on pathology	0	184 (100.0%)
History of prior positive temporal artery biopsy	33	23 (15.2%)

Values represent median (quartile 1, quartile 3) for continuous variables and frequency (percentage) for categorical variables. *NYHA*, New York Heart Association.

Echocardiographic findings are shown in Table 2. Median maximum size of ascending aorta, arch, and descending thoracic aorta on preoperative echocardiogram was 57 mm (IQR, 54, 62), 35 mm (IQR, 30, 41), and 31 mm (IQR, 27, 39), respectively. Moderate or greater aortic regurgitation was present in 62 (54.0%) patients. Significant aortic stenosis was not present in any patient. There were 15 (8.1%) patients who had previous surgery performed at outside hospitals before their index operation at the Mayo Clinic.

**Table 2:** Preoperative echocardiogram findings in patients with giant cell arteritis

	Number missing	Overall (N = 184)
Ejection fraction, %	66	62 (56, 66)
Annulus diameter, mm	84	23 (21, 25)
Sinus of Valsalva diameter, mm	81	38 (36, 43)
STJ diameter, mm	115	35 (30, 39)
Maximum ascending aorta diameter, mm	69	57 (54, 62)
Maximum arch diameter, mm	111	35 (30, 41)
Maximum descending aorta diameter, mm	144	31 (27, 39)
Aortic regurgitation	69	
Moderate		41 (35.7%)
Severe		21 (18.3%)

Values represent median (quartile 1, quartile 3) for continuous variables and frequency (percentage) for categorical variables. *STJ*, Sinotubular junction.

Primary indication for surgery was aneurysm in 179 (97.3%), aortic dissection in 4 (2.2%), and coronary artery disease in 1 (0.5%) patient. There were 16 (8.7%) patients who underwent nonelective surgery (Table 3).

**Table 3:** Operative characteristics in patients with giant cell arteritis

	Overall (N = 184)
Primary indication	
Aneurysm	179 (97.3%)
Dissection	4 (2.2%)
CAD	1 (0.5%)
Surgery number	
1	169 (91.8%)
2	14 (7.6%)
3	1 (0.5%)
Nonelective surgery	16 (8.7%)
Ascending aortic procedure	184 (100.0%)
Aortic root procedure	46 (25.0%)
Arch aorta procedure - hemi	109 (59.2%)
Arch aorta procedure - total	30 (16.3%)
Frozen elephant procedure	6 (4.4%)
Elephant trunk procedure	18 (13.1%)
Descending aortic procedure	7 (3.8%)
Thoracoabdominal aorta procedure	2 (1.1%)
Aortic valve replacement	25 (13.6%)
Aortic root reconstruction with valved conduit (Bentall)	23 (12.5%)
Combined operations	
CABG + valve + aorta	23 (12.5%)
CABG + aorta	24 (13%)
Valve + aorta	88 (47.8%)
Aorta	49 (26.6%)
Perfusion time, min	146 (106, 185)
Crossclamp time, min	87 (53, 123)
Circulatory arrest used	139 (75.5%)
Circulatory arrest time, min	17 (0, 24)

Values represent median (quartile 1, quartile 3) for continuous variables and frequency (percentage) for categorical variables. *CAD*, Coronary artery disease; *CABG*, coronary artery bypass grafting.

### Operative Procedure

In this study, our focus was on patients who underwent proximal aortic surgery attributable to GCA, and the most common indication was aneurysmal degeneration (97%). All patients underwent ascending aorta replacement. Approximately 59% and 16% of the patients underwent a hemiarch and a total arch replacement, respectively. Further extension into the arch was determined by evidence of any aneurysmal degeneration, adjacent vasculitis or on occasions as part of an open distal to have good-quality tissue without any calcification, or evidence of inflammatory changes. The surgical procedures are listed in Table 3. Cardiopulmonary bypass was initiated via arterial cannulation of the ascending aorta in 137 (74.5%) patients, axillary artery in 32 (17.4%), and femoral artery in 15 (8.2%) patients. Median cardiopulmonary bypass time was 146 minutes (IQR, 106, 185), and crossclamp time was 87 minutes (IQR, 53, 123). There were 139 (75.5%) patients who underwent deep hypothermic circulatory arrest for arch reconstruction with a median time of 17 minutes (IQR, 0, 24).

### Early Outcomes

Early mortality was 1.6% (n = 3). Postoperative complications are listed in Table 4. In brief, 8 (4.4%) patients required re-exploration for bleeding, 6 (3.3%) patients developed stroke, 3 (1.6%) patients developed new renal failure requiring dialysis, and 3 (1.6%) patients developed postoperative sternal infection (superficial or deep).

**Table 4:** Postoperative characteristics in patients with giant cell arteritis

	Number missing	Overall (N = 184)
In-hospital death	1	3 (1.6%)
30-d death	1	2 (1.1%)
Operative death	1	3 (1.6%)
Complication: reoperation for bleeding	1	8 (4.4%)
Complication: pneumonia	1	8 (4.4%)
Complication: prolonged ventilation	1	25 (13.7%)
Complication: sternal infection, superficial or deep	1	3 (1.6%)
Complication: stroke	1	6 (3.3%)
Complication: renal failure	1	7 (3.8%)
Complication: dialysis required	1	3 (1.6%)
Length of ICU stay, h	35	27 (21, 50)
Length of hospital stay, d	0	7 (6, 9)
Readmission within 30 d of surgery	8	20 (11.4%)

Values represent median (quartile 1, quartile 3) for continuous variables and frequency (percentage) for categorical variables. *ICU*, Intensive care unit.

Median length of intensive care unit stay was 27 hours (IQR, 21, 50) and hospital stay was 7 days (IQR, 6, 9). Twenty (11.4%) patients were readmitted within 30 days of surgery, mostly for cardiovascular complications.

**Figure 1:**
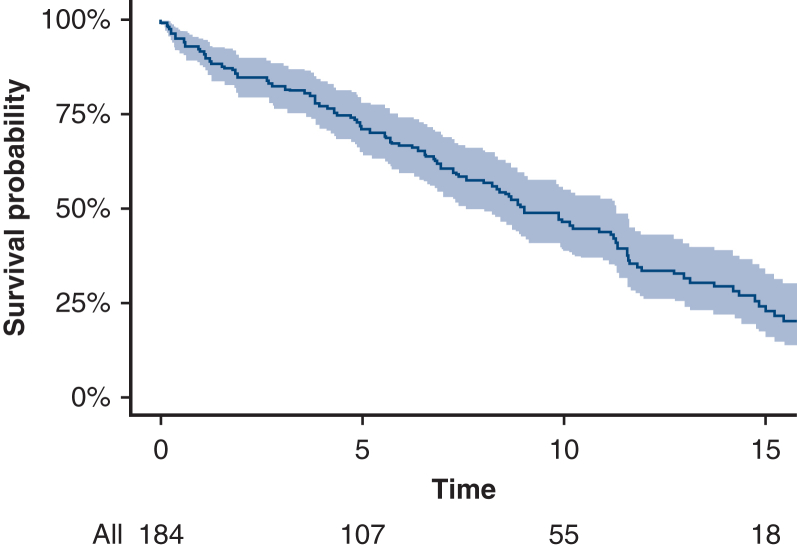
Kaplan-Meier curve of all-cause mortality in patients with giant cell arteritis (*shaded region* is 95% confidence limits). The median follow-up time using the reverse Kaplan-Meier method was 13.7 years. The median time to death using the Kaplan-Meier method was 9.03 years.

**Figure 2:**
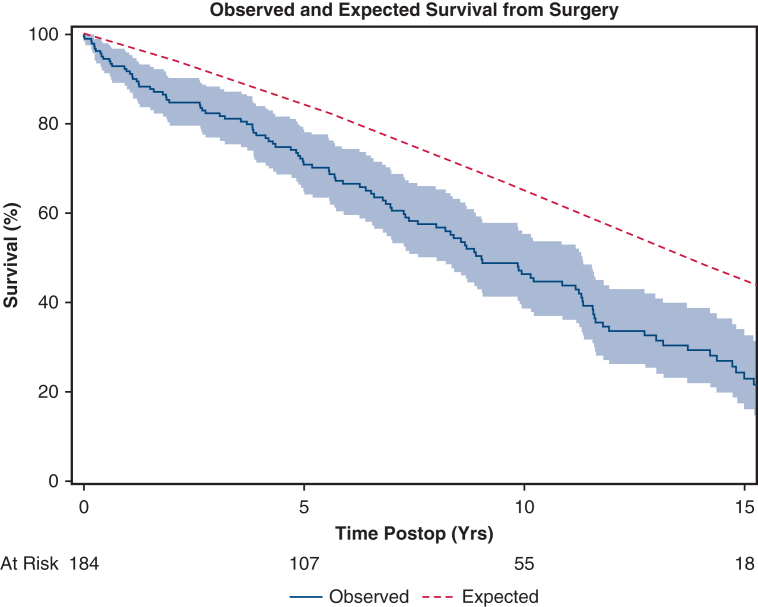
Observed and expected survival from surgery.

The median follow-up duration was 13.7 years (IQR, 5.8, 18.4). The median time to death was 9.0 years (IQR, 4.4, 14.8). Overall survival was 91.7% (95% CI, 87.7%-95.8%), 70.8% (95% CI, 64.1%-78.1%), 46.3% (95% CI, 38.7%-55.3%), and 24.2% (95% CI, 17.3%-33.9%) at 1, 5, 10, and 15 years, respectively (Figure 1). Multivariable analysis found advanced age (HR, 1.054; 95% CI, 1.026-1.082, *P* < .001), congestive heart failure within 2 weeks of surgery (HR, 1.890; 95% CI, 1.016-3.516, *P* = .04), history of peripheral vascular disease (HR, 2.229; 95% CI, 1.458-3.624, *P* < .001), and cerebrovascular disease (HR, 1.762; 95% CI, 1.035-3.000, *P* = .03) as predictors of late mortality (Table 5). The observed survival was worse compared with the expected survival of the White population in Minnesota with equivalent age and sex distribution (Figure 2).

**Table 5:** Multivariable Cox proportional hazard model of all-cause mortality in patients with giant cell arteritis

	HR	CI lower HR	CI upper HR	*P* value
Age	1.054	1.026	1.082	<.001
Sex female vs male	1.384	0.885	2.163	.154
Congestive heart failure within 2 wk yes vs no	1.890	1.016	3.516	.044
Peripheral vascular disease yes vs no	2.299	1.458	3.624	<.001
Cerebrovascular disease yes vs no	1.762	1.035	3.000	.037
Operative category CAB + valve + aorta vs aorta	1.096	0.557	2.159	.790
Operative category CAB + aorta vs aorta	1.306	0.723	2.359	.376
Operative category valve + aorta vs aorta	0.866	0.535	1.403	.560
Circulatory arrest used yes vs no	1.072	0.690	1.667	.756

*HR*, Hazard ratio; *CI*, confidence interval; *CAB*, coronary artery bypass.

### Reoperation

**Figure 3:**
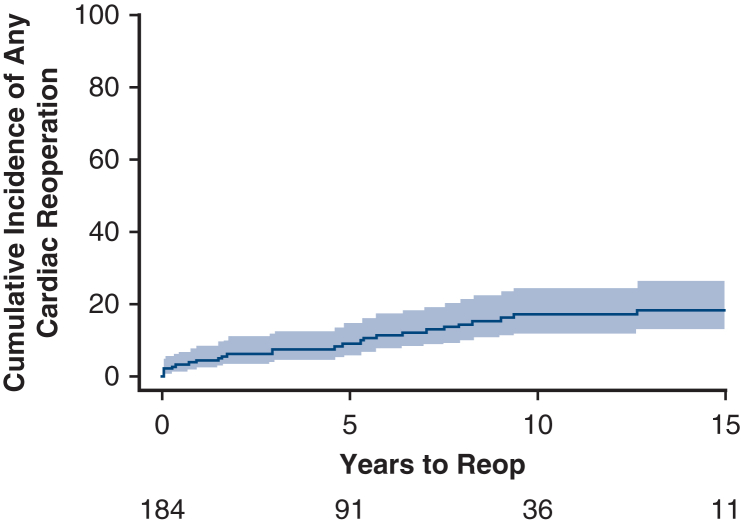
Cumulative incidence curve of all reoperations accounting for the competing risk of death (*shaded region* is 95% confidence limits). Cumulative incidence (time to first cardiac reoperation).

**Figure 4:**
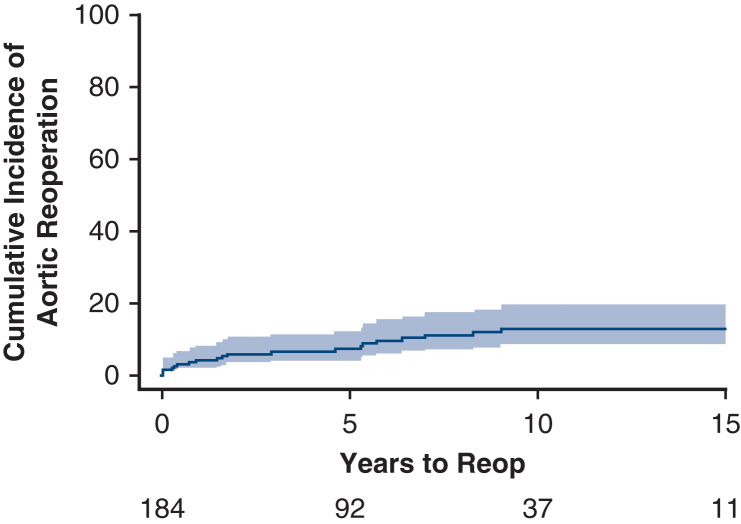
Cumulative incidence curve of aortic reoperations accounting for the competing risk of death (*shaded region* is 95% confidence limits). Cumulative incidence (time to first aortic reoperation).

**Figure 5:**
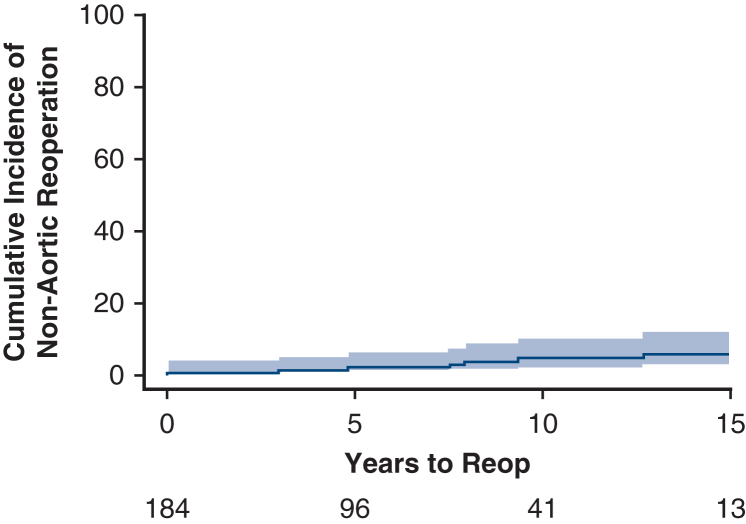
Cumulative incidence curve of nonaortic cardiac reoperations accounting for the competing risk of death (*shaded region* is 95% confidence limits). Cumulative incidence (time to first nonaortic cardiac reoperation).

Cumulative incidence of cardiac reoperation was 18.2% (95% CI, 12.7%-26.0%) at 15 years (Figure 3). A total of 32 reoperations occurred during the follow-up period on 26 patients. Twenty-two patients had only 1 reoperation, and 1 patient had 4 reoperations. We divided the reoperations into aorta-related and nonaorta, cardiac-related reoperations. Risk of aortic-related reoperation was 3.9% (95% CI, 1.9%-8.1%), 7.1% (95% CI, 4.1%-12.3%), 12.8% (95% CI, 8.3%-19.6%), and 12.8% (95% CI, 8.3%-19.6%), at 1, 5, 10, and 15 years, respectively (Figure 4). Risk of nonaorta cardiac-related reoperation was 0.5% (95% CI, 0.1%-3.9%), 1.9% (95% CI, 0.6%-5.9%), 4.5% (95% CI, 2.0%-9.9%), and 5.5% (95% CI, 2.7%-11.5%), at 1, 5, 10, and 15 years, respectively (Figure 5).

A total of 24 aorta-related reoperations were performed (Table E1). Index operation for all these patients were done in an elective manner. The most common indications for aorta-related reoperation were aneurysm (n = 16, 66.7%), rupture (n = 3, 12.5%), and anastomotic pseudoaneurysm (n = 2, 8.3%). The most common reoperation performed was open thoracoabdominal aortic aneurysm repair (n = 10, 41.7%). Eighteen (75.0%) aorta-related reoperations were done open and 6 were done through an endovascular approach (thoracic endovascular aortic repair, n = 5, 20.8%, fenestrated endovascular aortic repair, n = 1, 4.2%). All these patients were started on steroid treatment after their index surgery. At the time of repeat operation, 13 of these patients were still receiving steroid therapy.

Overall, 14 (58.3%) patients did not have an aortic specimen for pathology review after reoperation. From the 10 available aortic specimens, 2 had active GCA, 2 revealed healed aortitis, and 6 showed no GCA.

## Discussion

In the present work, we describe 184 patients who underwent ascending aorta replacement for aortic aneurysm and were found to have GCA on histopathologic evaluation of the aortic specimen. These patients were mostly asymptomatic at presentation. Hemiarch reconstruction was undertaken in the majority of patients. Our data demonstrate advancing age, heart failure, and peripheral arterial disease as risk factors for late mortality. Risk of future aorta-related reoperation was around 13% at 10 years, mostly involving descending aorta.

The median age of presentation, female preponderance, and approximately 30% overlap with PMR in the present study is consistent with previous studies^11^^,^^12^ and similar to how patients with classic cranial GCA present.^13^

However, in terms of symptomatology, only a minority of patients (3%-7%) in these series presented with symptoms attributable to cranial GCA such as headache, visual changes, and jaw claudication. This suggests that in these patients the branch vessels may be spared, unlike patients with classic cranial GCA, where the disease process demonstrates a certain tissue tropism favoring the involvement of branches of the external carotid artery.^14^ Chest pain and/or dyspnea on exertion were also uncommon symptoms in the present series and might have contributed to imaging studies that lead to identification of the aneurysm. Because most patients are asymptomatic from an aneurysm standpoint, a low threshold for thoracic imaging in patients with suspicion for GCA/PMR may not be unreasonable. Especially since it has been shown that in patients with a diagnosis of cranial GCA who have a normal aorta at index imaging, there is a less than 1% chance of developing aortic aneurysm at 5 years, but this risk increases to 12% at 10-year follow-up with median time to development of aortopathy of 7 years.^3^ The 10-year risk of developing aortopathy in patients with cranial GCA is greater than in the non-GCA population.^3^ Therefore, it may be prudent to consider a standardized approach for imaging surveillance at regular intervals in patients with newly diagnosed GCA.^3^

In the present study, the inflammatory process with evidence of aneurysmal degeneration involving the aortic arch required partial or total arch replacement in 109 (59.2%) and 30 (16.3%) patients at index surgery, respectively. Variable degree of similar aortic involvement distal to the ascending aorta has been reported previously.^15^^,^^16^ It is to be noted that, similar to previous studies, no evidence of aortic valve involvement was found in this study as well.

When the diagnosis of GCA is known before surgery, we prefer to perform the procedure with the patient in the state of remission. A thorough preoperative work-up is undertaken to assess disease activity. This includes careful clinical assessment to look for cranial and/or constitutional symptoms, biochemical evaluation with serial erythrocyte sedimentation rate and C-reactive protein laboratory testing, and imaging studies such as computed tomography scan, magnetic resonance imaging, and/or fluorodeoxyglucose F-18/positron emission tomography (FDG-PET). If the disease is active, glucocorticoid therapy is initiated and, depending on the response, steroid-sparing agents such as methotrexate and tocilizumab may be added. Typically, we wait for a period of 4 to 6 months to allow the disease activity to subside before taking up the patient for surgery.

However, if the diagnosis was made postsurgery from a pathologic specimen, we work in close association with our rheumatology colleagues to help manage these patients. Most commonly, we start the patient on glucocorticoid therapy in the immediate perioperative period (prednisone 1 mg/kg/d up to a maximum of 80 mg). We then follow these patients with serial clinical, biochemical (erythrocyte sedimentation rate and C-reactive protein levels) and imaging (computed tomography scan, magnetic resonance imaging, and/or FDG-PET) surveillance every 3 to 6 months. Depending on the response to treatment, further tapering of steroids is initiated (decrease of 20 mg every few weeks) or addition of other immunosuppressive agents such as tocilizumab, methotrexate, abatacept or other glucocorticoid sparing agents is made. If the clinical suspicion of relapse is high with an unequivocal biochemical evaluation, we use FDG-PET imaging to help guide our management. A long-term follow-up is important, as despite a quiescent disease state, further aortic intervention for aneurysmal degeneration may be needed, as shown by 24 patients who required reoperation in this study, with nearly one half of them being on preoperative steroids. Indeed, in this study from the 10 aortic specimens we had after reoperation, 80% did not show any evidence of active disease, perhaps suggesting that there may not be a direct correlation with disease activity and aneurysmal degeneration. After the second surgery, the goal was to assess for any ongoing disease activity and, if suggested so by clinical, biochemical, imaging and/or pathologic specimen, further immunosuppressive treatment was initiated, which might include restarting steroids if they were stopped earlier, or increasing the dose and adding further steroid-sparing agents.

Four (2%) patients in our cohort presented with acute type A aortic dissection. The incidence of concomitant GCA and aortic dissection has been reported to be less than 2%.^3^^,^^17^ Whether the inflammatory process of GCA predisposes the ascending aorta to dissection is unknown. There are also some reported cases of nonaneurysmal aortic rupture in patients with active giant cell aortitis.^18^ Data are scarce, and no conclusion can be drawn regarding treatment or surgical management. Nevertheless, this reflects perhaps screening of at least thoracic aorta in patients with newly diagnosed GCA might be beneficial.

In the present study, early outcomes were satisfactory, and further multivariable analysis found that advanced age, recent heart failure, peripheral vascular disease, and cerebrovascular disease were risk factors for late death. Operative category was not identified as a risk factor for lower survival. More than one half of our patients had moderate or greater aortic insufficiency, and in the absence of any aortic cusp–related vasculitis, this was most likely related to the aneurysmal dilatation of the aorta. Perhaps earlier diagnosis and surgical intervention may alter the survival by addressing the valve-related heart failure in this group of patients. Peripheral arterial disease was identified as another risk factor for poor long-term survival (HR, 2.30). It has been suggested that peripheral arterial disease may be a surrogate for a more advanced systemic vasculitis or occurs secondary to accelerated atherosclerosis in a setting of a chronic inflammatory process.^19^ It is also considered to be indicative of increased comorbidity burden.^20^ These likely explains the worse long-term prognosis seen in these patients.

In a study by Kebed and colleagues,^3^ the aneurysmal thoracic aorta in patients with GCA who did not undergo surgery yet was found to be increasing in size by 1.3 mm/year, which was greater than in those with degenerative aortic aneurysms (1.0 mm/year). There are no specific guidelines for surveillance of patients with aortic GCA after resection of the diseased aorta, and the fate of the remaining aorta is not clear. The present study shows that the risk of aorta-related reoperation in patients with a diagnosis of aortic GCA is 13% at 10 years, with aneurysmal degeneration of the remaining aorta (67%) being the most common indication. This suggests that resection of the diseased aorta in patients with GCA is not curative, as evidenced by a varying degree of aortitis on reoperative specimens found in our study. Therefore, it may not be unreasonable to have a surveillance protocol for these patients, likely similar to atherosclerotic aneurysmal disease. Further investigations and more data are also required to assess the duration of treatment of patients after aortic resection.

One of the indications for reoperation was development of anastomotic pseudoaneurysm. In case of a known diagnosis of GCA, we perform the procedure when the disease activity has subsided. However, in a setting in which the diagnosis is not known, we carefully evaluate for any inflammatory changes that can be seen in the aorta and excise all that aorta that may reflect such an appearance (cobblestone, mucoid, or thinned-out aorta with a lackluster look and some calcification) and find a healthy tissue to sew to. Some surgeons also use Teflon felt as an additional buttress, whereas we prefer to telescope the graft into the aorta as the anastomosis is performed. This we believe helps in achieving better hemostasis and providing some internal buttress.

### Limitations

This study evaluates a single-institution surgical cohort of patients in a 27-year period and has inherent limitations typical of a retrospective study. There was also lack of data on duration of concomitant medical management and the likely compliance with it. Although long-term follow-up was available in the majority of patients (95%), it is possible that reoperations at outside centers may have been missed. Lastly, given our practice represents a tertiary referral center, there could be an inherent selection bias.

## Conclusions

GCA presenting with ascending aorta aneurysm typically involves elderly patients with a female preponderance and is mostly asymptomatic. The early and late outcomes from proximal aorta surgery are satisfactory with low mortality. Advancing age, recent heart failure, peripheral vascular disease, and cerebrovascular disease are risk factors for worse survival, with descending aorta aneurysmal degeneration being the most common indication for reoperation. A low threshold for thoracic aorta imaging at the time of initial diagnosis of GCA and a standardized surveillance imaging approach may be considered. An earlier intervention before the onset of heart failure may improve survival.

## Conflict of Interest Statement

The authors reported no conflicts of interest.

The *Journal* policy requires editors and reviewers to disclose conflicts of interest and to decline handling or reviewing manuscripts for which they may have a conflict of interest. The editors and reviewers of this article have no conflicts of interest.

## Figures and Tables

**Table E1:** Aortic reoperations

	First reoperation (n = 19)	Second reoperation (n = 3)	Third reoperation (n = 1)	Fourth reoperation (n = 1)	Total (N = 24)
Primary indication					
Anastomotic pseudoaneurysm	2 (10.5%)	0 (0.0%)	0 (0.0%)	0 (0.0%)	2 (8.3%)
Aneurysm	14 (73.7%)	2 (66.7%)	0 (0.0%)	0 (0.0%)	16 (66.7%)
Endoleak	0 (0.0%)	0 (0.0%)	1 (100.0%)	0 (0.0%)	1 (4.2%)
IMH	0 (0.0%)	1 (33.3%)	0 (0.0%)	0 (0.0%)	1 (4.2%)
Infection	0 (0.0%)	0 (0.0%)	0 (0.0%)	1 (100.0%)	1 (4.2%)
Rupture	3 (15.8%)	0 (0.0%)	0 (0.0%)	0 (0.0%)	3 (12.5%)
Redo operation					
Arch-DTA bypass	0 (0.0%)	0 (0.0%)	0 (0.0%)	1 (100.0%)	1 (4.2%)
Total arch (ET)r	1 (5.3%)	0 (0.0%)	0 (0.0%)	0 (0.0%)	1 (4.2%)
Total arch (ET)r + TEVAR	1 (5.3%)	0 (0.0%)	0 (0.0%)	0 (0.0%)	1 (4.2%)
Total arch (FE)r	1 (5.3%)	0 (0.0%)	0 (0.0%)	0 (0.0%)	1 (4.2%)
DTAr	3 (15.8%)	0 (0.0%)	0 (0.0%)	0 (0.0%)	3 (12.5%)
TEVAR	2 (10.5%)	2 (66.7%)	1 (100.0%)	0 (0.0%)	5 (20.8%)
FEVAR	1 (5.3%)	0 (0.0%)	0 (0.0%)	0 (0.0%)	1 (4.2%)
PSA repair	1 (5.3%)	0 (0.0%)	0 (0.0%)	0 (0.0%)	1 (4.2%)
TAAAr	9 (47.4%)	1 (33.3%)	0 (0.0%)	0 (0.0%)	10 (41.7%)
Aortic involvement					
Arch	3 (15.8%)	0 (0.0%)	0 (0.0%)	0 (0.0%)	3 (12.5%)
Arch + descending	1 (5.3%)	0 (0.0%)	0 (0.0%)	0 (0.0%)	1 (4.2%)
Descending	5 (26.3%)	2 (66.7%)	1 (100.0%)	1 (100.0%)	9 (37.5%)
Thoracoabdominal	10 (52.6%)	1 (33.3%)	0 (0.0%)	0 (0.0%)	11 (45.8%)
Time to reoperation, y					
Median (Q1, Q3)	1.74 (0.55, 5.53)	2.87 (1.55, 4.95)	3.39 (3.39, 3.39)	8.70 (8.70, 8.70)	2.89 (0.64, 5.88)

*IMH*, Intramural hematoma; *DTA*, descending thoracic aorta; *ET*, elephant trunk; *TEVAR*, thoracic endovascular aortic repair; *FE*, frozen elephant; *FEVAR*, fenestrated endovascular aortic repair; *PSA*, pseudoaneurysm; *TAAAr*, thoracoabdominal aortic aneurysm repair.

## References

[1] Machado E.B., Michet C.J., Ballard D.J. (1988). Trends in incidence and clinical presentation of temporal arteritis in Olmsted County, Minnesota, 1950-1985. Arthritis Rheum.

[2] Maleszewski J.J. (2015). Inflammatory ascending aortic disease: perspectives from pathology. J Thorac Cardiovasc Surg.

[3] Kebed D.T., Bois J.P., Connolly H.M. (2018). Spectrum of aortic disease in the giant cell arteritis population. Am J Cardiol.

[4] Miller D.V., Isotalo P.A., Weyand C.M., Edwards W.D., Aubry M.C., Tazelaar H.D. (2006). Surgical pathology of noninfectious ascending aortitis: a study of 45 cases with emphasis on an isolated variant. Am J Surg Pathol.

[5] Nuenninghoff D.M., Hunder G.G., Christianson T.J.H., McClelland R.L., Matteson E.L. (2003). Incidence and predictors of large-artery complication (aortic aneurysm, aortic dissection, and/or large-artery stenosis) in patients with giant cell arteritis: a population-based study over 50 years. Arthritis Rheum.

[6] Bossert M., Prati C., Balblanc J.C., Lohse A., Wendling D. (2011). Aortic involvement in giant cell arteritis: current data. Joint Bone Spine.

[7] Homme J.L., Aubry M.C., Edwards W.D. (2006). Surgical pathology of the ascending aorta: a clinicopathologic study of 513 cases. Am J Surg Pathol.

[8] Liang K.P., Chowdhary V.R., Michet C.J. (2009). Noninfectious ascending aortitis: a case series of 64 patients. J Rheumatol.

[9] Espitia O., Samson M., Le Gallou T. (2016). Comparison of idiopathic (isolated) aortitis and giant cell arteritis-related aortitis. A French retrospective multicenter study of 117 patients. Autoimmun Rev.

[10] Talarico R., Boiardi L., Pipitone N. (2014). Isolated aortitis versus giant cell arteritis: are they really two sides of the same coin?. Clin Exp Rheumatol.

[11] Zehr K.J., Mathur A., Orszulak T.A., Mullany C.J., Schaff H.V. (2005). Surgical treatment of ascending aortic aneurysms in patients with giant cell aortitis. Ann Thorac Surg.

[12] Rojo-Leyva F., Ratliff N.B., Cosgrove D.M., Hoffman G.S. (2000). Study of 52 patients with idiopathic aortitis from a cohort of 1,204 surgical cases. Arthritis Rheum.

[13] Salvarani C., Gabriel S.E., O’Fallon W.M., Hunder G.G. (1995). The incidence of giant cell arteritis in Olmsted County, Minnesota: apparent fluctuations in a cyclic pattern. Ann Intern Med.

[14] Gravanis M.B. (2000). Giant cell arteritis and Takayasu aortitis: morphologic, pathogenetic and etiologic factors. Int J Cardiol.

[15] Prieto-González S., Arguis P., García-Martínez A. (2012). Large vessel involvement in biopsy-proven giant cell arteritis: prospective study in 40 newly diagnosed patients using CT angiography. Ann Rheum Dis.

[16] Gagné-Loranger M., Dumont É., Voisine P. (2016). Giant cell aortitis: clinical presentation and outcomes in 40 patients consecutively operated on. Eur J Cardiothorac Surg.

[17] Richardson M.P., Lever A.M., Fink A.M., Dixon A.K., Hazleman B.L. (1996). Survival after aortic dissection in giant cell arteritis. Ann Rheum Dis.

[18] Eklund E., Englund E., Valdemarsson S. (1998). Rupture of a non-aneurysmatic aortic trunk in a patient with giant cell arteritis. Ann Rheum Dis.

[19] Ungprasert P., Thongprayoon C., Kittanamongkolchai W., Srivali N., Cheungpasitporn W. (2016). Peripheral arterial disease in patients with giant cell arteritis: a meta-analysis. Int J Rheum Dis.

[20] Li L., Neogi T., Jick S. (2017). Giant cell arteritis and vascular disease-risk factors and outcomes: a cohort study using UK Clinical Practice Research Datalink. Rheumatology (Oxford).

